# A disease, disorder, illness or condition: How to label epilepsy?

**DOI:** 10.1111/ane.12757

**Published:** 2017-03-14

**Authors:** A. J. Noble, A. Robinson, A. G. Marson

**Affiliations:** ^1^ Department of Psychological Sciences University of Liverpool Liverpool UK; ^2^ Department of Molecular and Clinical Pharmacology University of Liverpool Liverpool UK

**Keywords:** epilepsy, language expression, perception, stigma

## Abstract

The International League Against Epilepsy (ILAE) is an important source of guidance for health professionals when it comes to epilepsy. Their latest recommendation that epilepsy should no longer be called a “disorder,” but a “disease” has though caused controversy. The ILAE contends the change will improve epilepsy's image. Some clinicians and other organizations fear the change may not though be accepted by patients as in common parlance “disease” can be associated with “contagiousness”/”infection.” To allow practicing clinicians to make informed judgements about what language they use, we completed the first study to assess the preferences of those with epilepsy and significant others and explore if any of their characteristics were associated with preference. Via epilepsy interest groups and associations in England, Wales, Scotland and the Republic of Ireland, 971 patients and significant others were surveyed. Participants identified which of four labels for epilepsy (“disorder,” “illness,” “disease,” “condition”) they favoured and rated each using a Likert‐scale. Patients’ median age was 39; 69% had experienced seizures in the prior year. “Condition” was favoured by most patients (74.3%) and significant others (71.2%). Only 2.2% of patients and 1.2% of significant others chose “disease”; it received a median Likert‐rating indicating “strongly dislike.” Multinomial logistic regression found it was not possible to reliably distinguish between participants favouring the different terms on the basis of demographics. The ILAE's position is at odds with what most patients and carers want and we discuss the implications of this.

## 1 INTRODUCTION

The International League Against Epilepsy (ILAE) is an important source of guidance when it comes to epilepsy.^1^ Their recently published revised, operational definition of epilepsy 2 is therefore noteworthy. A key change is that epilepsy is no longer a “disorder,” rather it is now to be labelled a “disease.” This is intended to improve epilepsy's profile. “Disorder,” they said, was “poorly understood by the public and implied a functional, temporary disturbance and so minimised the seriousness of epilepsy” (p.476).^2^ “Disease” was felt to hold more gravity and thus better communicate epilepsy's life implications.^3^

Epilepsy's profile within the public domain does need improving. Attitudes towards epilepsy are often negative and it attracts less attention than its prevalence and impact warrant.^4^, ^5^ There is no evidence that calling epilepsy a disease will address this. Marketing experts have though recommended it: “[Y]ou cannot arouse public passion about a ‘disorder’, you have to call epilepsy a ‘disease’”^6^ (p.2363).

Some though, have advised against the change.^7^, ^8^ When the ILAE's draft went for consultation, 113 comments relating to the change were submitted.^7^ Health professionals were concerned it would not be supported by patients and carers. They said “disease” remains poorly defined and, within common parlance, is often associated with ideas of “contagiousness” and “infectiousness.”^7^ It was also contended the label may have ramifications for how someone accepts a new diagnosis and views themselves.

Whilst the label change is clearly contentious amongst health professionals, it is not actually known how patients and carers view it. Their preferences have not been systematically examined. To allow clinicians and others to make informed decisions about the language they adopt when discussing epilepsy, we asked a large sample of persons living with epilepsy and significant others about label preferences. We also tested which, if any, of their characteristics were associated with their preference.

## 2 METHODS

### 2.1 Participants

Persons with epilepsy and significant others (i.e., friends, family, informal carers). All were aged ≥18 years. Patients were those self‐reporting a clinical diagnosis of epilepsy (any syndromes/seizure type). People were excluded if they could not provide informed consent or were unable to independently complete English questionnaires.

### 2.2 Procedure

Between December 2015 and February 2016, an online survey was conducted. It was designed to address wider questions regarding perceptions of stigma.^9^, ^10^ Participants were recruited by advertisements in the newsletters and websites of epilepsy interest groups and organizations within England, Scotland, Wales and the Republic of Ireland. People wishing to take part were directed to an online survey page.

The University of Liverpool's Institute of Psychology Health and Society Research Ethics Committee approved the study (IPHS‐1516‐SMc‐105). Informed consent was obtained from all.

### 2.3 Measures

#### 2.3.1 Characteristics

Participants reported their demographics and medical history. Table 1 details the information asked for.

**Table 1:** Participant characteristics

	Patients (N=638)	Family and friends (N=333)
Age		
Median (IQR)	39 (28‐49.25)	46 (39‐55)
20‐31 y	198 (31.0)	46 (13.8)
32‐42 y	168 (26.3)	80 (24.0)
43‐51 y	142 (22.3)	98 (29.4)
52‐81 y	130 (20.4)	109 (32.7)
Sex (n/%)		
Female	489 (76.6)	298 (89.5)
Male	149 (23.4)	35 (10.5)
Ethnicity (n/%)		
White British	604 (94.7)	319 (95.8)
Other	34 (5.3)	14 (4.2)
Main spoken language		
English	626 (98.1)	327 (98.2)
Other	12 (1.9)	6 (1.8)
Confidence in English if not first language		
“Very well”	5 (41.7)	5 (83.3)
“Well”	7 (58.3)	1 (16.7)
“Not well”	0 (0.0)	0 (0.0)
“Not at all”	0 (0.0)	0 (0.0)
Highest educational attainment (n/%)		
Basic school certificate or lower	169 (26.5)	85 (25.5)
Advanced school certificate or equivalent	142 (22.3)	66 (19.8)
University degree, Diploma or higher	327 (51.3)	182 (54.7)
Employment (n/%)		
Employed (full/part‐time)/student	389 (61.0)	217 (65.2)
Homemaker/Other	172 (27.)	108 (32.4)
Unemployed	77 (12.1)	8 (2.4)
Main epilepsy doctor (n/%)		
Primary care	142 (22.3)	‐
Hospital specialist	371 (58.2)	
Equally shared between primary care and specialist	125 (19.6)	
Age at diagnosis		
Median (IQR)	18 (12‐27)	‐
Years diagnosed		
Median (IQR)	16 (7‐28)	‐
Anti‐epileptic medication (n/%)		
None	28 (4.4)	‐
Monotherapy	272 (42.6)	
Polytherapy	338 (53.)	
Seizures (any type) prior 12 mo^a^ (n/%)		
Yes	441 (69.1)	‐
No	197 (30.9)	
Median (IQR)	5.0 (0‐10)	
Experience convulsive seizures? (n/%)		
Yes	478 (74.9)	‐
No	160 (25.1)	
Nocturnal seizures only? (n/%)		
No	546 (85.6)	‐
Yes	92 (14.4)	
Reported cause of epilepsy		
Unknown	429 (67.2)	‐
Acquired brain injury	90 (14.1)	
Other	119 (18.7)	
Medical history (beyond epilepsy; n/%)		
None	356 (55.8)	‐
Another medical diagnosis	202 (31.7)	
Psychiatric diagnosis	32 (5.0)	
Both medical and psychiatric diagnoses	45 (7.5)	
Relationship to patient.		
Spouse/partner	‐	40 (12.0)
Parent		234 (70.3)
Friend		24 (7.2)
Child		11 (3.3)
Other^b^		24 (7.2)

IQR, interquartile range; n,number; SD, standard deviation.

a Thapar et al.'s scale asks “How many attacks have you had in the last 12 mo?” The participant can choose from the following ordinal categories: 0, 1, 2, 3, 4, 5, 6, 7, 8, 9, 10 or more.

b Other category includes siblings, cousins, aunts and uncles.

#### 2.3.2 Preference

Participants were presented with the following: “People use different terms/expressions to describe what epilepsy is. For example, some people call epilepsy a ‘condition’, whereas others call it an ‘illness’. Below are some sentences that use different terms.” They then saw four different phrases: “Epilepsy is an illness,” “Epilepsy is a condition,” “Epilepsy is a disease,” and “Epilepsy is a disorder.” Participants identified which one term they preferred and rated the extent to which they liked each using a Likert‐scale (1=“strongly dislike,” 5=“strongly like”).

### 2.4 Analysis

Descriptive statistics were produced using STATA 11 (Stata Corporation, College Station, TX, USA). Proportions with 95% confidence intervals (95% CI) and medians, along with the interquartile range (IQR) are reported to describe participants’ preferences. Patients’ and significant others’ preferences were analysed separately.

Multinomial logistic regression, with robust standard errors, explored whether any of the participants’ characteristics were associated with label preference. Only a small number of participants ultimately selected “disease” and its inclusion in predictive modelling would have made the resulting models unstable. It was therefore excluded and regression completed with marginally smaller samples to determine how participant characteristics were associated with preference for any of the three remaining labels. Unadjusted regression was first completed. Any significantly associated (*P*<.05) variables were then simultaneously entered into adjusted regression analyses to identify parsimonious predictors. Where a cell of a categorical independent variable contained fewer than five participants, the characteristic was not examined (see notes to Tables S1 and S2). Relative risk ratios (RRR) along with 95% CIs describe associations. To determine how well the final models predicted preference, area under the curve (AUC) statistics were estimated using the “mlogitroc” command, which generates multiclass ROC curves for classification accuracy using bootstrapping methods and smoothed probability distributions derived from kernel density estimation.^11^An AUC of 0.5 represents random prediction, whilst an AUC of 1 represents perfect prediction.

## 3 RESULTS

### 3.1 Participant characteristics

One thousand and eighty‐two participants were recruited (695 patients and 387 significant others). Of these, 89.7%, n=971 (638 patients and 333 significant others) had complete preference data and were included in analyses (Table 1).

There were no statistical differences between patients with and without complete data in age, sex or ethnicity (all *P*>.05). Significant others with and without missing data also did not differ from one another in age or ethnicity. Males were though, more likely to have missing data (23.9% vs 12.6%; *P*=.03).

Patients’ median age was 39 (IQR=28‐49.25), and 76.6% were female. Median years since diagnosis was 16 (IQR=7‐28). Most (69.1%) had experienced a seizure within the prior year. Median age of significant others was 46 (IQR=39‐55), and most were female (89.5%). Most (70.3%) were a parent to someone with epilepsy.

### 3.2 Preference

Most patients (n=474; 74.3%; 95% CI: 70.9‐77.7) and significant others (n=237; 71.2%; 95% CI: 66.3‐76.1) chose “condition” as the term they favoured (Table 2). The median rating it received from patients and significant others on the preference scale was 4, equating to “Like.”

**Table 2:** Preferred terms and ratings for what epilepsy should be labelled as

Term	Patients (N=638)			Significant others (N=333)		
	Most favoured term		Preference rating	Most favoured term		Preference rating
	n (%)	95% CI	Median (IQR)	n (%)	95% CI	Median (IQR)
Epilepsy is an condition	474 (74.3)	70.9‐77.7	4 (4‐5)	237 (71.2)	66.3‐76.1	4 (4‐4)
Epilepsy is an illness	90 (14.1)	11.4‐16.8	2 (1‐3)	50 (15.0)	11.2‐18.9	3 (2‐3)
Epilepsy is a disorder	60 (9.4)	7.1‐11.7	3 (1‐3)	42 (12.6)	9.0‐16.2	3 (2‐4)
Epilepsy is a disease	14 (2.2)	1.1‐3.3	1 (1‐2)	4 (1.2)	0.0‐2.3	1 (1‐2)

IQR, interquartile range; CI, confidence interval; Higher scores on preference rating scale reflect greater preference (1=“strongly dislike,” 2=“dislike,” 3=“neither like or dislike,” 4=“like,” 5=“strongly like”).

Only 14 (2.2%, 95% CI: 1.1‐3.3) patients and 4 (1.2%, 95% CI: 0.0‐2.3) significant others chose “disease” as their favoured term. The median preference rating given to it was 1, indicating “strongly dislike.”

### 3.3 Characteristics associated with label most favoured

In unadjusted analyses years diagnosed with epilepsy, the presence of comorbidity, employment status and sex were significantly associated with patients preference (Table S1). When entered into an adjusted model, only the presence of a comorbidity and sex remained significant. Those with a comorbidity (n=36, 19.1%) were twice as likely to favour “illness” over “condition” than those without one (n=54, 10.4%; adjusted RRR=2.03, 95% CI: 1.26, 3.28), whilst females (n=39, 8.2%) were less likely to favour “condition” over “disorder” than males (n=21, 14.3%; adjusted RRR=0.52, 95% CI: 0.29, 0.92). The AUC statistic for this final model was 0.56, indicating poor overall predictive ability.

For significant others, their age, employment status and the number of seizures they estimated the person they knew to have experienced in the prior year were significantly associated with preference in unadjusted analyses (Table S2). In adjusted analyses, being currently employed or in education was associated with being more likely to prefer “disorder” over “condition” (adjusted RRR=2.37, 95% CI: 1.15, 4.86), as was an increase in seizures (adjusted RRR=1.12, 95% CI: 1.02, 1.24).) Again, the AUC statistic for this model, 0.50, indicated poor overall predictive ability.

## 4 DISCUSSION

### 4.1 Patient and carer preference

Our results indicate most patients and informal carers do not want epilepsy to be labelled a “disease” —9/10 are against it. “Condition” is instead favoured. This new evidence can be used by neurologists and others to help them decide what language to adopt.

To minimize participant burden, we did not ask participants’ the reasons for their preference. We did complete regression analyses to explore if preference was associated with the patient's or significant other's characteristic to see whether this could offer any insights. A handful of variables were associated with preference. Overall, however, the ability to use this information to reliably predict preference was limited. This suggests additional factors beyond those which we examined are important in determining preference. It might therefore be most helpful to consider the results of the current survey in the context of reasons given by persons with epilepsy in online forums as to why they object to the label “disease.”^12^ Patients there appear concerned that within lay use, “disease” is associated with “infectiousness” and that this could contribute to misconceptions. Indeed, this appears why no patient‐led epilepsy organization operating in an English speaking country has adopted the ILAE's new stance (Table S3.).

Whether “disease” does evoke more ideas of “contagiousness” in the minds of the public is not known. There are diagnoses where it does not appear to (e.g., coronary heart disease, Alzheimer's). However, might it in the context of epilepsy? If so, this would be concerning as older ideas about epilepsy remain and so terms reinforcing them need avoiding. Austin et al.^13^ found only half of American adolescents were confident epilepsy was not contagious.

### 4.2 Implications

The ILAE's Task Force acknowledged their definition was informed by available evidence. At the time, none existed on patients or carers’ preferences. Now it is, should their position change?

It could be reasoned that it should not. It was done with the admirable goal of improving epilepsy's image. If it ultimately does this, the change, however unpopular, would be justified. On the other hand, the ILAE themselves^2^ stated “A definition should conform to how clinicians and patients think…” (p.476). It appears the “disease” element of their revised definition does not align with what most patients or carers (and many clinicians) think. This could become a source of tension between the ILAE and those it seeks to support. One patient has already publically noted^7^:

It [epilepsy] is not contagious, although there are some who still believe it is. They think I have a disease, and avoid me so as not to catch it. Now some of the most eminent epileptologists on the planet are telling me that those ignorant and bigoted people are correct. Does this mean it's time for another identity crisis?

#### 4.2.1 Strengths and weaknesses

Strengths include that recruitment happened across multiple countries. Our large sample means study estimates also have narrow CIs.

Potential weaknesses are that our participants were recruited via newsletters from epilepsy organizations and interest groups. Not all people receive such correspondence and the views of these persons might differ from those who do. Our patients were also slightly younger and more educated than those in the wider epilepsy population. This likely occurred because participation was online; 86% of UK households have Internet access, but age and cost are barriers.^14^

Our findings may have international relevance as the study focused on English phrases. English is the third most widely first‐spoken language^15^ and the language of science. Our study does not, however, indicate what the preferences are of people when another language is used. Some of the phrases we presented to our participants do not have equivalents in some languages and even when they can be directly translated, it is unclear whether the terms have the same connotations as the English phrases that those with epilepsy and their carers appear concerned about.

## 5 CONCLUSION

Findings suggest the ILAE needs to better communicate its reasons for calling epilepsy a “disease” or reverse its decision. At present, labelling epilepsy a “disease” is at odds with what most patients and carers want.

## CONFLICT OF INTEREST AND SOURCES OF FUNDING STATEMENT

On behalf of all authors, the corresponding author states that there is no conflict of interest.
