# Supplementary material for: A disease, disorder, illness or condition: How to label epilepsy?
Source: Acta Neurol Scand. 2017 Mar 14;136(5):536–40. doi: 10.1111/ane.12757 (PMC5655763; doi:10.1111/ane.12757)
Supplement: Supplementary file 1 [file ANE-136-536-s001.docx]

**Supplementary Table 1** Patient characteristics which held a statistically significant unadjusted association with label preference and their adjusted association

| **Patients** (N=638) | | | |
| --- | --- | --- | --- |
| **Label preference** | **Variable** | **Unadjusted RRR (95% CI)** | **Adjusted RRR (95% CI)** |
| *Epilepsy is a condition* | Base condition | | |
| *Epilepsy is an illness* | Sex  Female  Male | 1.00 Reference  0.81 (0.48, 1.36) | 0.79 (0.47, 1.36) |
|  | Employment  Unemployed  Homemaker/ Other  Employed (full/ part-time)/ student | 1.00 Reference  1.21 (0.74, 1.99)  **0.56 (0.36, 0.89)** | 0.71 (0.44, 1.16) |
|  | Years diagnosed | **1.02 (1.002, 1.03)** | 1.01 (0.99, 1.03) |
|  | Medical history (beyond epilepsy)  Yes, at least one comorbidity  None | 1.00 Reference  **2.18 (1.38, 3.46)** | **2.03 (1.26, 3.28)** |
| *Epilepsy is a disorder* | Sex  Male  Female | 1.00 Reference  **0.52 (0.29, 0.92)** | **0.52 (0.29, 0.92)** |
|  | Employment  Unemployed  Homemaker/ Other  Employed (full/ part-time)/ student | 1.00 Reference  1.21 (0.67, 2.18)  0.60 (0.35, 1.04) | 0.69 (0.40, 1.22) |
|  | Years diagnosed | 1.01 (0.99, 1.03) | 1.01 (0.99, 1.03) |
|  | Medical history (beyond epilepsy)  Yes, at least one comorbidity  None | 1.00 Reference  1.56 (0.91, 2.67) | 1.48 (0.86, 2.54) |
| **Model** |  |  | **N= 624, *X*^2^ (8)= 26.14, *P<0.005*,**  **Pseudo *R*^2^ = 0.028** |

*Notes* Reference category: Epilepsy is a condition. RRR= Relative risk ratio; CI= confidence interval. Entries in bold indicate statistically significant differences (P<0.05).Variables examined for the association with label preference but which did not demonstrate a statistically significant unadjusted association were patient age, highest educational attainment, marital status, main epilepsy doctor (Hospital specialist, Primary care, Equally shared), age at diagnosis, antiepileptic treatment (polytherapy, monotherapy/ no medication), number of seizures (any type) in prior 12 months, experience convulsive seizures, and reported cause of epilepsy. Due to the small cell size it was not possible to test for the association between ethnicity, first spoken language or the experience of nocturnal seizures only and label preference.

**Supplementary Table 2** Family and friend characteristics which held a statistically significant unadjusted association with label preference and their adjusted association

| **Significant others** (N=329) | | | |
| --- | --- | --- | --- |
| **Preference category** | **Variable** | **Unadjusted RR (95% CI)** | **Adjusted RR (95% CI)** |
| *Epilepsy is a condition* | Base condition | | |
| *Epilepsy is an illness ^a^* | Age | 0.99 (0.97, 1.03) | 0.99 (0.97, 1.02) |
|  | Employment  Unemployed/Other  Employed (full/ part-time)/ student | 1.00 Reference  1.26 (0.67, 2.39) | 1.28 (0.68, 2.41) |
|  | Seizures (any type) prior 12 months ^a^ | 1.0004 (0.92, 1.08) | 0.99 (0.93, 1.08) |
| *Epilepsy is a disorder ^a^* | Age | **1.03 (1.01, 1.06)** | 1.02 (0.99, 1.05) |
|  | Employment  Unemployed/Other  Employed (full/ part-time)/ student | 1.00 Reference  **2.99 (1.53, 5.86)** | **2.37 (1.15, 4.86)** |
|  | Seizures (any type) prior 12 months ^a^ | **1.14 (1.03, 1.26)** | **1.12 (1.02, 1.24)** |
| **Model** |  |  | **N= 329, *X*^2^ (6)= 17.49, *P<*0.01**  **Pseudo *R*^2^ =0.038** |

*Notes* Reference category: Epilepsy is a condition. RRR= Relative risk ratio; CI= confidence interval. Entries in bold indicate statistically significant differences (P<0.05).Variables examined for the association with label preference but which did not demonstrate a statistically significant unadjusted association were significant others’ sex, highest educational attainment, marital status, relationship to patient and whether they lived with patient.

**Supplementary Table 3** How epilepsy is labelled by different epilepsy organisations operating in English speaking countries

| **Country** | **Organisation** | **Definition of epilepsy** |
| --- | --- | --- |
| **United States** | | |
|  | Epilepsy Foundation ^a^ | “Epilepsy is a condition of the brain causing seizures”  “Epilepsy is a neurological condition which affects the nervous system.” |
| **Australia** | | |
|  | Epilepsy Australia Ltd ^h^ | “Epilepsy is a disorder of brain function that takes the form of recurring convulsive or non-convulsive seizures.” |
|  | Epilepsy Action Australia ^i^ | “Epilepsy is a common neurological condition affecting up to 1-2% of the population.” |
|  | Epilepsy Queensland ^j^ | “Epilepsy is a tendency to have recurring seizures… Epilepsy is one of the oldest conditions known …” |
| **South Africa** | | |
|  | Epilepsy South Africa ^m^ | “It is a neurological condition…It is not a psychological disorder, disease or illness and it is not contagious.” |
| **Canada** | | |
|  | Canadian Epilepsy Alliance/alliance Canadienne De Lʹepilepsie^k^ | “Epilepsy is a condition of the brain characterized by recurrent seizures…It is not a psychological disorder or a disease…” |
| **New Zealand** | | |
|  | Epilepsy Association Of New Zealand Inc ^l^ | “Epilepsy is a common neurological disorder affecting….” |
| **Singapore** | | |
|  | Singapore Epilepsy Foundation | “Epilepsy is not a disease, and it is not contagious. It is a common neurological disorder that causes sudden, uncontrollable electrical surges in the brain.” |
| **United Kingdom & ROI** | | |
|  | Epilepsy Scotland ^b^ | “Epilepsy is the most common, serious neurological disorder in the world…. It is not contagious, nor is it a disease. Between seizures the brain works normally. |
|  | Epilepsy Action ^c^ | “Epilepsy is a condition that affects the brain.” |
|  | SUDEP Action ^d^ | “Epilepsy is a condition in the brain. “ |
|  | Epilepsy Society ^e^ | “Epilepsy is a common serious neurological condition where there is a tendency to have seizures that start in the brain.” |
|  | Epilepsy Connections ^f^ | “Epilepsy is a common neurological condition that affects 1 in 103 people in the UK.” |
|  | Epilepsy Ireland ^g^ | “Epilepsy is the most common serious neurological disorder, affecting people of all ages.” |

*Notes:* National epilepsy organisations identified as International Bureau for Epilepsy organisations from <http://www.ibe-epilepsy.org/about/ibe-chapters/> (last accessed 2/10/16); Information on how epilepsy is labelled by the organisations was obtained from the following sources which were last accessed on 19/09/2016: ^a^ <http://www.epilepsy.com/learn/about-epilepsy-basics>; ^b^ <http://www.epilepsyscotland.org.uk/what-is-epilepsy-/info_13.html>; ^c^ <https://www.epilepsy.org.uk/info/what-is-epilepsy>; ^d^ <https://sudep.org/knowing-your-epilepsy>; ^e^ <https://www.epilepsysociety.org.uk/what-epilepsy#.V-AjfbFwazl>; ^f^ <http://www.epilepsyconnections.org.uk/wp-content/uploads/2013/07/Epilepsy-an-introduction.pdf>; ^g^ <http://www.epilepsy.ie/assets/48/C5448F49-563A-4502-952ACF89223BDAE9_document/Explaining_Epilepsy.pdf>; ^h^ <http://www.epilepsyaustralia.net/epilepsy-explained/>; ^i^ <http://www.epilepsy.org.au/about-epilepsy/understanding-epilepsy>; ^j^ <http://www.epilepsyqueensland.com.au/about-epilepsy-1>; ^k^ <http://epilepsysupport.ca/seizure-education/about/epilepsy>; ^l^ <http://epilepsy.org.nz/faq>; ^m^ <http://epilepsy.org.za/new/uploads/files/Fact%20sheets%202013/49821FactsaboutEpilepsy.pdf>; ^n^ <http://www.epilepsy.com.sg/epilepsy.html>
